# Balance Training Enhances Vestibular Function and Reduces Overactive Proprioceptive Feedback in Elderly

**DOI:** 10.3389/fnagi.2017.00273

**Published:** 2017-08-11

**Authors:** Isabella K. Wiesmeier, Daniela Dalin, Anja Wehrle, Urs Granacher, Thomas Muehlbauer, Joerg Dietterle, Cornelius Weiller, Albert Gollhofer, Christoph Maurer

**Affiliations:** ^1^Department of Neurology and Neurophysiology, University Hospital Freiburg Freiburg, Germany; ^2^Institute for Sports and Sport Science, University of Freiburg Freiburg, Germany; ^3^Department of Internal Medicine, Institute for Exercise and Occupational Medicine, University Hospital Freiburg Freiburg, Germany; ^4^Division of Training and Movement Science, University of Potsdam Potsdam, Germany; ^5^Division of Movement and Training Sciences, Biomechanics of Sport, Institute of Sport and Movement Sciences, University Duisburg-Essen Essen, Germany

**Keywords:** age, balance, vestibular, proprioception, training

## Abstract

**Objectives:** Postural control in elderly people is impaired by degradations of sensory, motor, and higher-level adaptive mechanisms. Here, we characterize the effects of a progressive balance training program on these postural control impairments using a brain network model based on system identification techniques.

**Methods and Material:** We analyzed postural control of 35 healthy elderly subjects and compared findings to data from 35 healthy young volunteers. Eighteen elderly subjects performed a 10 week balance training conducted twice per week. Balance training was carried out in static and dynamic movement states, on support surfaces with different elastic compliances, under different visual conditions and motor tasks. Postural control was characterized by spontaneous sway and postural reactions to pseudorandom anterior-posterior tilts of the support surface. Data were interpreted using a parameter identification procedure based on a brain network model.

**Results:** With balance training, the elderly subjects significantly reduced their overly large postural reactions and approximated those of younger subjects. Less significant differences between elderly and young subjects' postural control, namely larger spontaneous sway amplitudes, velocities, and frequencies, larger overall time delays and a weaker motor feedback compared to young subjects were not significantly affected by the balance training.

**Conclusion:** Balance training reduced overactive proprioceptive feedback and restored vestibular orientation in elderly. Based on the assumption of a linear deterioration of postural control across the life span, the training effect can be extrapolated as a juvenescence of 10 years. This study points to a considerable benefit of a continuous balance training in elderly, even without any sensorimotor deficits.

## Introduction

Impairments of postural control result in increased rates of unintentional falls. In fact, falls are the leading cause of injuries and subsequent deaths among people 65 years and older, and generate a fundamental financial burden to the healthcare system (Burns et al., 2016). There is general consensus that altered postural control in elderly people is determined by degradations of the sensory channels, i.e., vestibular, visual, and proprioceptive cues (Rauch et al., 2001; Goble et al., 2009; Grossniklaus et al., 2013), of the motor system (Macaluso and De Vito, 2004), and by deficits in higher-level adaptive systems (Shumway-Cook and Woollacott, 2001). It is still under debate whether, in addition, elderly's central weighting of sensory signals is affected. While some authors reported an impaired sensory weighting (Teasdale and Simoneau, 2001; Eikema et al., 2014), others found it to be unimpaired (e.g., Allison et al., 2006; Jeka et al., 2006). This controversy is possibly caused by different experimental strategies to assess sensory weighting. For example, it is well known that sensory weighting is modified by e.g., type and size of external disturbances, available sensory information, training status etc. (Oie et al., 2002; Peterka, 2002; Maurer et al., 2006). In general, it is unclear which subsystem mainly determines the degradation of postural control, given the fact that many subsystems are altered during aging.

From a diagnostic side, postural control is often monitored via spontaneous sway measures and, more rarely, challenged by external perturbations leading to motor reactions. Some authors reported age-related changes in spontaneous sway in terms of increased mean velocity, or increased sway frequencies (Prieto et al., 1996; Qu et al., 2009). However, the diagnostic value of spontaneous sway measures has been questioned (Maurer and Peterka, 2005; Pasma et al., 2014). For a more detailed analysis of postural control, the application of external perturbations has frequently been suggested. Interestingly, postural reactions to external perturbations (proprioceptive, vestibular, or visual) have been reported to be altered in the elderly (e.g., Ghulyan et al., 2005; Maitre et al., 2013; Eikema et al., 2014). More recently, the relationship between stimulus and subsequent body motion was systematically evaluated using model simulations (e.g., Peterka, 2002; Davidson et al., 2011; Nishihori et al., 2011; van der Kooij and Peterka, 2011). Models are usually based on simple feedback mechanisms, involving inverted pendulum bodies, stiffness, damping, feedback time delay, and sensory weighting (Maurer et al., 2006; van der Kooij and Peterka, 2011; Engelhart et al., 2014; Wiesmeier et al., 2015). They have already been applied to elderly people's postural control (Maurer and Peterka, 2005; Cenciarini et al., 2010; Davidson et al., 2011; Nishihori et al., 2011). Some authors reported increased damping of the system in the elderly (Cenciarini et al., 2010; Davidson et al., 2011). Stiffness findings are inconsistent (Maurer and Peterka, 2005; Cenciarini et al., 2010; Davidson et al., 2011; Nishihori et al., 2011). Surprisingly, systematic evaluations of intervention programs like balance training are completely lacking.

The improvement of elderly people's postural control via balance training is well documented (see e.g., Nagy et al., 2007; Gillespie et al., 2012). However, evidence for an optimal training program of healthy elderly people is scarce (Lesinski et al., 2015). Recently, Lesinski et al. (2015) concluded from a systematic review and metaanalysis of numerous training studies that an optimal training should last 11–12 weeks with a training frequency of three sessions per week resulting in a total number of 36–40 training sessions. A single training session should take 31–45 min. Over the last few years, balance training has been further diversified into traditional, perturbation-based, and multitask balance training approaches (see e.g., Granacher et al., 2011). A growing body of literature deals with the specific neurophysiological effects of balance training. Balance training may be able to reduce coactivation of antagonist muscles, to shorten onset latency of muscle activation, to augment reflex activity, to increase maximal and explosive force production capacity, increase the length of recovery steps subsequent to external perturbations (see Granacher et al., 2011). On a functional level, gait speed and step length, with or without external perturbations, have been reported to be increased, while step time variability seems to be reduced. Performance in clinical tests like Berg Balance Scale (BBS) and Timed Up and Go (TUG) test appears to be improved. This improvement was backed up by electrophysiological correlates, such as, the reduction of the Hoffmann reflex (Granacher et al., 2011; Nagai et al., 2012). However, it is unclear as yet, how balance training affects physiological subsystems of postural control, such as, use of sensory input, central processing, and motor output.

In the current study, we aimed to assess the main subsystems of elderly people's altered postural control with a focus on their sensitivity to balance training using parameter identification techniques based on brain network model simulations. We expected that balance training could change elderly subjects' postural control so that it resembles postural control of younger subjects, similar to a “juvenescence.”

## Methods

**Figure 1:**
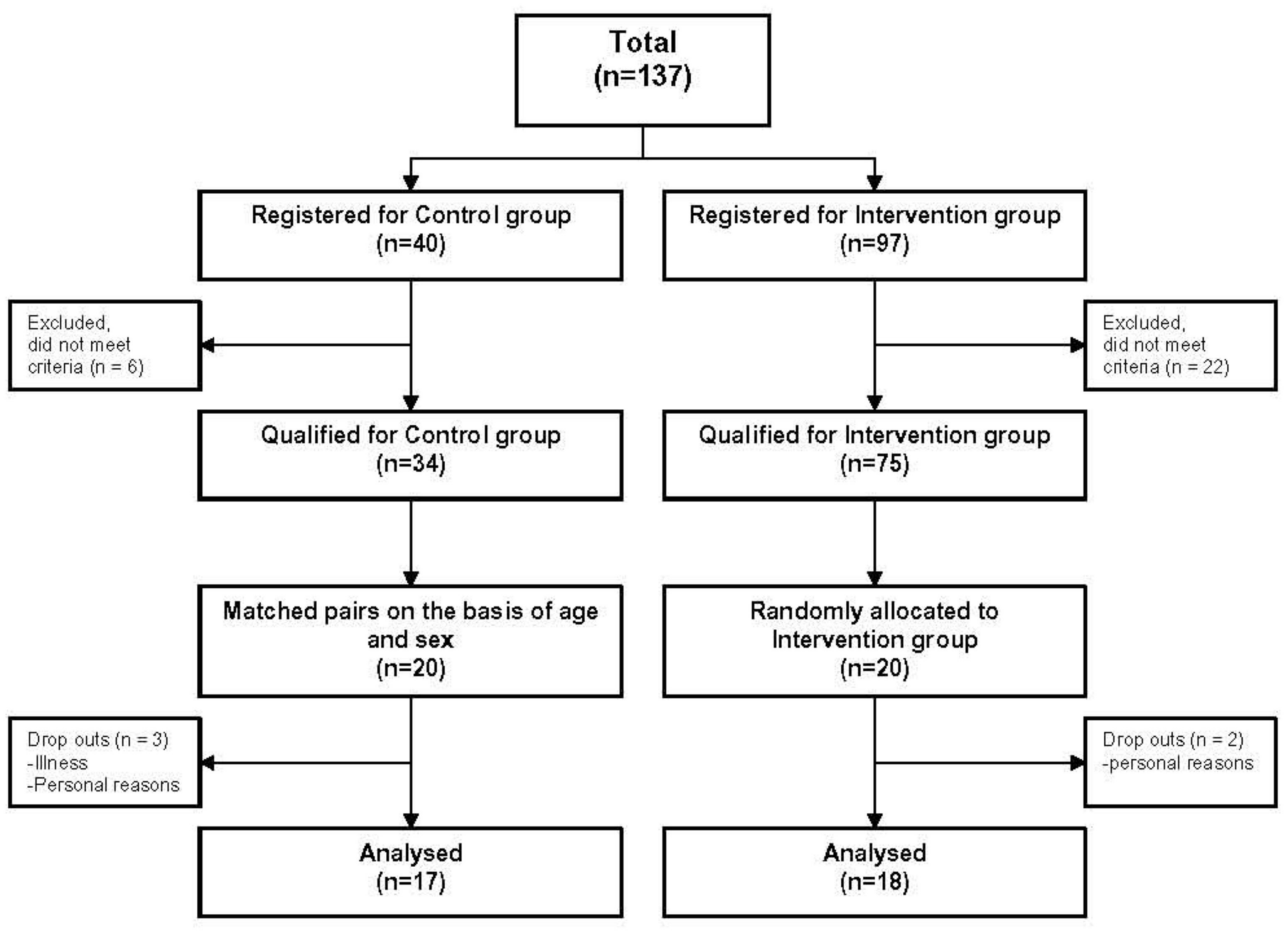
Flow chart of the study. 18 subjects of the training and 17 subjects of the control group were analyzed. Their data was compared with data of 35 younger subjects.

Forty elderly subjects between 65 and 80 years who lived independently in the community, were randomly allocated either into a balance training or into a control group that did not receive balance training. Allocation followed a matched-pair protocol on the basis of age and sex (see Figure 1). Each subject was examined by a senior consultant neurologist in order to identify sensory deficits or neurodegenerative diseases. In addition, we asked for the amount of physical activity, fear of falling and number of falls during the last 3 years prior to the study (for questionnaire, see Supplementary Material). As part of the neurological examination, vestibular function was specifically tested using Frenzel goggles on a turning chair (vestibulo-ocular reflex, VOR). Proprioceptive function was evaluated by testing position sense and by measuring vibration sense with a tuning fork. Elderly subjects with relevant sensory deficits were excluded from the study. Moreover, subjects suffering from any other acute or chronic disease that may interact with the postural control were excluded. Finally, 35 elderly subjects [73 ± 3.3 years (mean age ± *SD*)] contributed to the study.

In order to identify age-related changes in elderly subjects' postural control, we used younger subjects' data [*n* = 35, 37 ± 11.2 years (mean age ± *SD*)] generated with a similar experimental set in our laboratory, as a reference group. The study was approved by the ethics committee of the University of Freiburg and performed according to the ethical standards of the Declaration of Helsinki. Written informed consent was obtained from all subjects prior to study participation.

**Figure 2:**
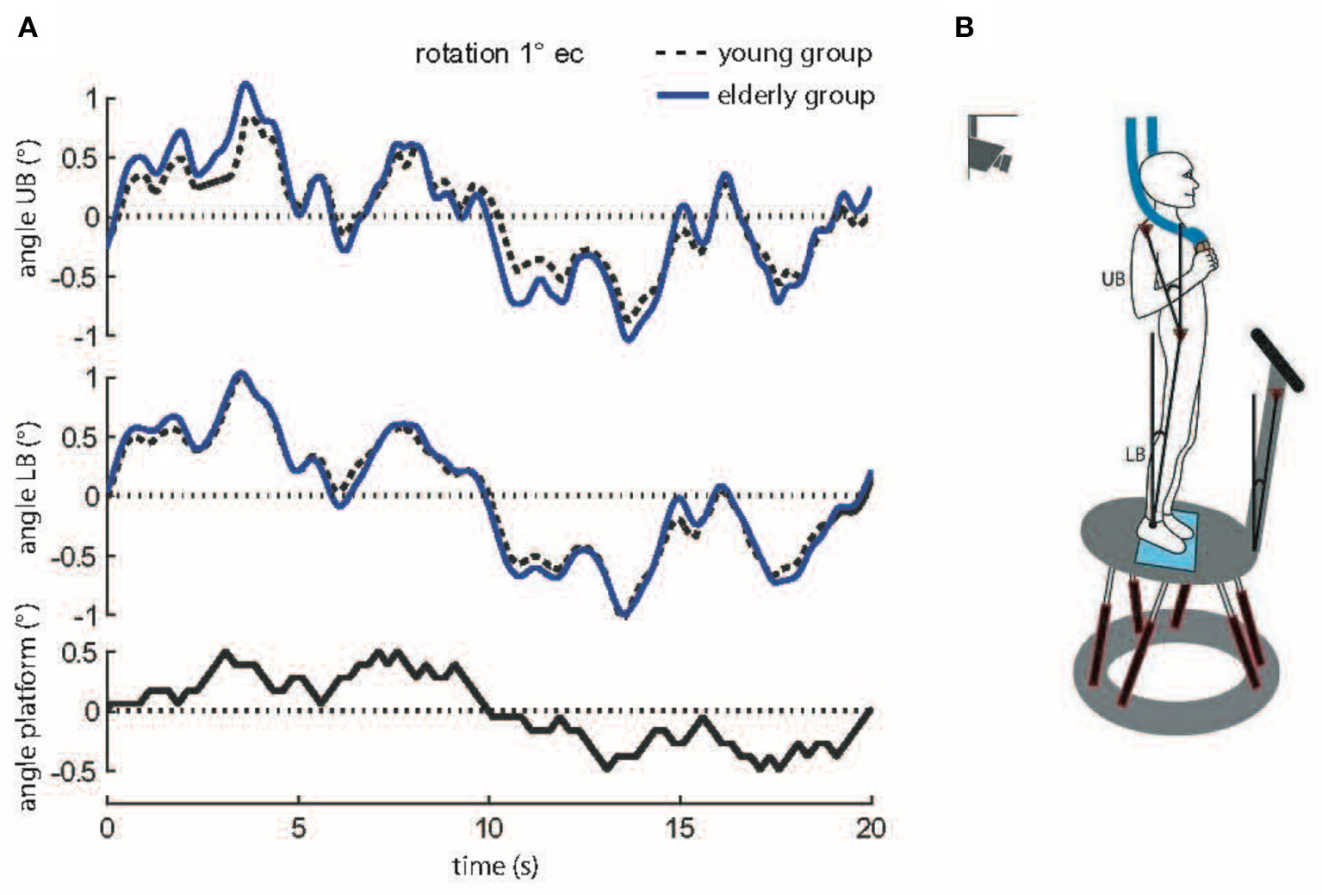
**(A)** Motor reactions of the mean body excursions of young and elderly subjects in relation to platform tilts. Shown are motor reactions in degrees of the upper body (angle UB), lower body (angle LB), and platform movements (sample stimulus, black bottom line). The traces of the young and elderly groups represent means across all subjects at 1° stimulus amplitude with eyes closed (s, seconds). **(B)** Experimental setup. Picture of a subject standing on the platform. For safety reasons subjects held ropes in their hands which hung loosely from the ceiling. The six linear motors created platform tilts with the axis running through the ankle joints. Angular excursions of the platform, the upper body (UB) and the lower body (LB) in space were quantified with an optoelectronic device using markers attached to shoulder, hip, and a rigid bar on the platform.

For evaluating postural control we used a dynamic posturography approach. Subjects were standing on a custom-built motion platform (Figure 2B) with eyes open (eo) and with eyes closed (ec). Spontaneous sway was recorded with the platform fixed while postural reactions were measured during continuous platform perturbations. Posturographic assessments were composed of 20 trials divided into two sessions: During the first 10 trials subjects were told to close their eyes while the other 10 trials were carried out with eyes open. The first and last trial of each ten-trial sequence (eyes closed or eyes open) was a ‘spontaneous sway’ trial. The other eight trials were conducted while the platform tilted. Each trial took 1 min. Breaks of about 10 s were taken between trials, according to the subject's needs. Subjects were told to stand comfortably in an upright position. They were asked not to talk.

Spontaneous sway was quantified by center-of-pressure (COP) sway paths detected with the help of a force transducing platform (Kistler platform type 9286, Winterthur, Switzerland). Extracted measures consisted of sway amplitude (Root Mean Square, RMS), sway velocity (Mean Velocity, MV), and the frequency content of sway (Mean Frequency, MF). Postural reactions were measured on a tilting platform. The tilts consisted of platform rotations in the sagittal plane with the axis running through subjects' ankle joints. Platform tilts were designed as pseudorandom stimuli (PRTS, pseudorandom ternary sequence, Figure 2A) with two peak angular displacements (0.5 and 1°) and analyzed at 11 frequencies (0.05, 0.15, 0.3, 0.4, 0.55, 0.7, 0.9, 1.1, 1.35, 1.75, and 2.2 Hz).

Angular excursions of the platform and the body (hip-to-ankle, shoulder-to-hip) in space were quantified with an optoelectronic device using markers attached to shoulder, hip, and a rigid bar on the platform (Optotrak 3020, Waterloo, Canada). Each marker contained three light-emitting diodes (LEDs). 3-D LED positions were used to calculate marker movements (Figures 2A,B). Kistler® and Optotrak® output as well as the stimulus signals were sampled at 100 Hz using an analog-digital converter and stored on a PC via LabView® (National Instruments, Austin, Texas, USA) for offline analysis. Data was analyzed using custom-made software programmed in MATLAB® (The MathWorks Inc., Natick, MA, USA).

The relationship between the postural reactions and platform stimuli were represented by “transfer functions” in the frequency domain. Transfer functions were calculated using discrete Fourier transforms. From transfer functions, GAIN, PHASE, and Coherence values were extracted as a function of stimulus frequencies. GAIN represents the size of the postural reaction, i.e., lower body or upper body response in terms of angular excursion, as a function of stimulus size (platform angle). A GAIN of 1 would indicate a perfect match between body and platform excursion. PHASE is related to the relative timing between postural reaction and stimulus. Negative PHASE values (PHASE lag) represent delays. Coherence is a measure for reproducibility of postural reactions across stimulus cycles. Coherence values of 1 signify perfectly reproduced postural reactions; zero would indicate no similarity between subsequent postural reactions.

Findings in the elderly were compared with data of a young reference group. In addition, data of the elderly group before (first assessment, A1) was compared to data after balance training (second assessment, A2). The second assessment was conducted between 4 and 10 days following the last training session.

In addition, the TUG and the Functional Reach Test (FRT) were assessed twice (A1 and A2) in elderly subjects. The TUG quantifies the time (in seconds), a subject needs to do the following motor task: standing up from a chair, walking 3 m straight, turning around, walking back, and sitting down. The FRT measures the maximum distance in centimeters before and after reaching the arm forward at shoulder level without losing balance (Enkelaar et al., 2013). Mean FRT was calculated across three attempts.

### Parameter identification

**Figure 3:**
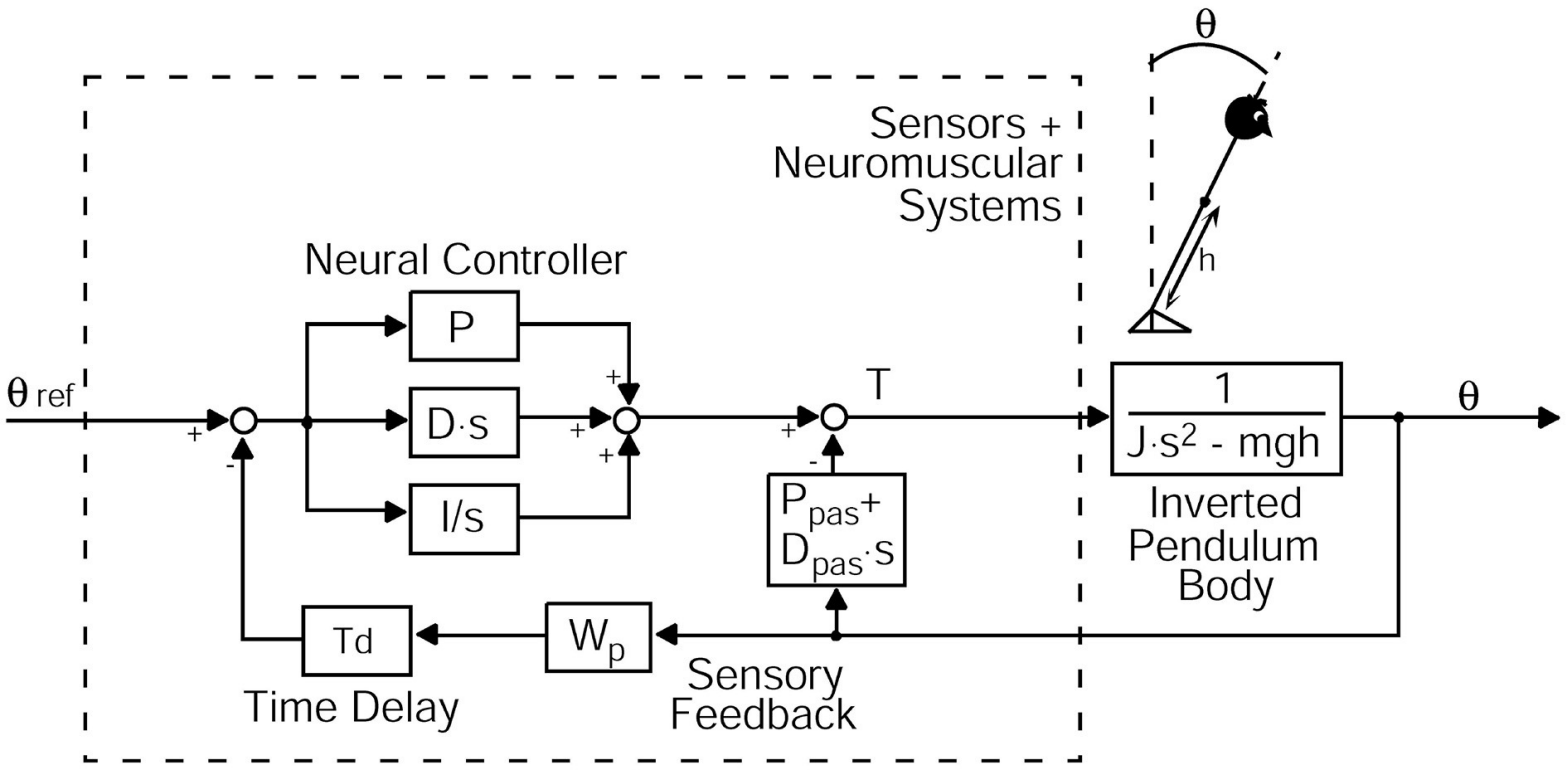
Model simulation describing perturbed stance. The model includes a body in terms of an inverted pendulum with the sensory and neuromuscular systems including a Neural Controller. The mass is concentrated at the center of mass (COM) of the body. Θ, body sway angle; h, height of the COM above the ankle joints; Θ ref, external stimulus; P, proportional gain (stiffness factor), D, derivative gain (damping factor), I, integral gain of the Neural Controller; Ppas, passive stiffness factor; Dpas, passive damping factor; Wp, proprioceptive sensory weight; Td, feedback time delay; T, control torque; J, moment of inertia of the body; mgh, body mass·gravitational constant·height of the COM from the ankle joint; s, Laplace transform variable.

Transfer functions served as a basis for simulating postural control using well-established models of upright stance to extract relevant parameters (Peterka, 2002; Engelhart et al., 2014). The model includes a body defined by mass and height, a Neural Controller containing stiffness and damping, a feedback time delay, and a sensory feedback mechanism. A negative feedback loop links body excursions perceived by visual, vestibular, and proprioceptive channels to a corrective torque through a Neural Controller with proportional [P], derivative [D] and integral [I] contributions (PDI-controller, Figure 3). The external stimuli, i.e., anterior-posterior platform tilt angles, serve as an input of the model. Body sway, represented by the center of mass (COM) angle, is the model output. Since Neural Controller values depend on mass and height of the individual subjects (see Peterka, 2002; Cenciarini et al., 2010), these values are corrected by (mgh), which corresponds to the gravitational pull (body mass) × (gravitational constant) × (height of the COM from the ankle joint), leading to [P/mgh], [D/mgh], and [I/mgh]. Other parts of the model were: a lumped time delay [Td], representing the time interval between the postural reaction and the stimulus, and a sensory weighting mechanism. The sensory weighting mechanism specifies the reference frame for body orientation (space coordinates vs. platform coordinates), represented by [Wp]. The value [Wp] stands for the proprioceptive share of the sensory feedback. A value of 1 corresponds to 100 % proprioceptive control, i.e., stabilization in platform coordinates, a value of 0 relates to 0% proprioceptive control and 100% stabilization in space. Moreover, the model includes a biomechanics part that represents torque related to passive elasticity [Ppas] and damping [Dpas] of muscles and tendons, in parallel to the active corrective torque, which is determined by the Neural Controller (Figure 3). With the help of an optimization procedure (fmincon/ Matlab, Mathworks), model simulations were fitted to the experimental transfer functions under different stimulus amplitudes and visual conditions. Similar to the model provided by Peterka (2002), we assume that all sensory feedback signals add up to a feedback gain of unity. For example, if the proprioceptive gain is 0.6 (60%) in the eyes-closed condition, the vestibular gain would be 0.4 (40%). In the eyes-open condition, vestibular and visual gains both contribute to the space reference. The strength of the visual feedback could be estimated by subtracting the gain of the space reference in the eyes-closed condition from the space reference in the eyes-open condition. Another assumption refers to the allowed range of all gains ([P/mgh], [D/mgh], [I/mgh], [Wp], [Ppas], [Dpas]), and time delay [Td], which were constrained to positive values. Goodness-of-fit measures, limitations of the model, and comparisons to simulations of datasets from other studies are provided as Supplementary Material.

### Balance training program

The balance training group received a balance training twice a week over a period of 10 weeks (one session 60 min, 20 sessions in total). It was developed and conducted by professional instructors from the Institute for Sports and Sport Science (University of Freiburg) and the Institute for Exercise- and Occupational Medicine (Department of Internal Medicine, University Hospital Freiburg) on the basis of previous research (Granacher et al., 2010). The training group was divided into two smaller exercise groups which were supervised by two instructors in order to guarantee a small participant-to-instructor ratio (1 instructor vs. 5 subjects). Each session included 10 min warm-up and 10 min cool-down. Exercises were carried out under static (standing) and dynamic (walking) conditions and were modified by using either a stable or an unstable support surface (e.g., foam mats), by closing or opening the eyes and by performing the exercises in bipedal, semi-tandem, tandem and monopedal stance with an additional motor task like catching and throwing a ball. One set of exercises consisted of 4 periods of 20 s exercise and 40 s rest. The number of sets was increased over time. Many exercises were performed in pairs or as circuit training.

### Statistical analysis

Statistical analyses were performed using Microsoft Excel and statistic programs (JMP® and Statview by SAS Institute Inc., Cary, NC, USA). Statistical significance of the difference between healthy young and elderly subjects before training was tested with the help of an analysis of variance (ANOVA). The between-subject variable was group (young, elderly). For spontaneous sway, the within-subject variables were: visual condition (eyes open, eyes closed), sway direction (mediolateral, anteroposterior), and body segment (COP, hip, shoulder). For the perturbed stance experiments, the within-subject variables were: visual condition, stimulus amplitude (0.5 and 1°) and body segment (hip, shoulder). Intervention effects in the two groups of elderly subjects before (A1) and after (A2) balance training including FRT and TUG was tested by multivariate analyses of variance (MANOVA) with time (A1, A2) as an additional degree of freedom. Statistical significance was assumed at *p* ≤ 0.05. Moreover, the relationships between parameters related to platform measures and clinical test parameters were examined with a Pearson Correlation Test. A matrix of correlation coefficients was created, which illustrates the strength of linear relationships between each pair of parameters.

## Results

### Baseline characteristics

Thirty-five healthy elderly [73 ± 3.3 years (mean age ± *SD*), 17 female, 18 male] and 35 young subjects [37 ± 11.2 years (mean age ± *SD*), 19 female, 16 male] were included in the analysis. None of the subjects reported any training or test-related injuries. For detailed information see Tables 1, 2. Three subjects of the control group dropped out due to failure to attend the second assessment for personal reasons and illness. Two subjects of the training group dropped out during the training period due to personal reasons not associated with balance training. Both, the training and control groups were well balanced at baseline concerning age, sex, body mass, and physical activity (Table 2). In total, 11 subjects claimed to have fallen during the last 3 years prior to the study (training group: 6, control group: 5). The number of falls was similar in both groups (training group: 10 falls, control group: 7 falls). Reasons for falling were e.g., tripping or leisure time activities. Seven subjects reported fear of falling (training group: 4, control group: 3) which was always associated with particular situations such as, clear ice or standing on a ladder.

**Table 1:** Information about the young group.

**Subject number**	**Age [ys]**	**Body mass [kg]**	**Body height [m]**
1	20–25	#	1.67
2	25–30	40.5	1.63
3	25–30	97.0	1.69
4	30–35	90.3	1.80
5	20–25	#	1.58
6	25–30	#	1.63
7	30–35	73.5	1.79
8	25–30	72.5	1.82
9	20–25	66.0	1.80
10	20–25	43.0	1.60
11	30–35	79.8	1.78
12	25–30	79.3	#
13	25–30	72.5	1.78
14	30–35	#	1.90
15	25–30	82.0	1.84
16	30–35	76.0	1.83
17	25–30	92.0	2.03
18	20–25	74.5	1.64
19	20–25	66.0	1.79
20	45–50	#	1.67
21	40–45	77.0	1.75
22	40–45	58.5	1.80
23	55–60	83.3	1.72
24	40–45	88.8	1.90
25	50–55	67.8	#
26	45–50	60.3	1.60
27	55–60	82.8	1.70
28	45–50	#	1.76
29	45–50	50.3	1.68
30	45–50	#	1.56
31	45–50	97.0	1.78
32	45–50	50.8	1.72
33	45–50	53.8	1.66
34	50–55	65.0	1.66
35	35–40	#	1.69

*35 subjects; 19 female; 16 male. m, masculine; f, feminine; ys, years; kg, kilogram; m, meters; #, no data available*.

**Table 2:** Information about the training and control group.

**Subject number**	**Group**	**Age [ys]**	**Body mass [kg]**	**Body height [m]**	**Physical activity [h/week]**	**Fear of falling**	**Number of falls in the last 3 years**
1	Control	70–75	56.0	1.58	8.5	No	
2	Control	70–75	79.0	1.76	1.0	No	
3	Control	65–70	98.0	1.91	6.0	No	
4	Control	70–75	68.0	1.61	2.5	Yes	1
5	Control	75–80	71.5	1.71	0	No	
6	Control	70–75	55.0	1.52	14.0	No	2
9	Control	70–75	75.0	1.61	2.5	No	1
10	Control	75–80	61.5	1.57	5.0	Yes	
11	Control	65–70	95.0	1.72	4.0	No	
12	Control	70–75	94.5	1.86	8.5	No	
13	Control	75–80	73.0	1.67	12.5	No	
14	Control	70–75	80.5	1.83	5.0	No	1
15	Control	70–75	98.5	1.80	0.0	No	
16	Control	75–80	64.0	1.56	7.0	No	2
17	Control	65–70	80.0	1.76	2.0	No	
18	Control	75–80	67.0	1.58	2.0	No	
19	Control	75–80	60.5	1.62	4.0	Yes	
21	Training	80–85	68.0	1.63	1.0	Yes	3
23	Training	65–70	90.0	1.76	0.0	No	1
25	Training	65–70	64.5	1.51	9.0	No	
26	Training	70–75	79.0	1.74	6.0	Yes	
27	Training	65–70	90.5	1.77	10.0	No	2
28	Training	70–75	71.5	1.67	2.0	No	
29	Training	75–80	65.0	1.51	1.5	No	
30	Training	65–70	89.0	1.76	0	Yes	
31	Training	70–75	75.0	1.65	4.5	No	
32	Training	75–80	65.5	1.70	6.5	No	
33	Training	70–75	65.0	1.68	3.5	No	
34	Training	70–75	81.5	1.78	4.0	No	
35	Training	70–75	64.5	1.67	0	No	
36	Training	75–80	86.0	1.71	4.5	No	
37	Training	70–75	70.0	1.66	10.5	No	1
38	Training	70–75	88.5	1.75	4.0	Yes	
39	Training	75–80	70.0	1.52	1.0	No	1
40	Training	75–80	71.0	1.73	6.5	No	2

*35 subjects; 17 female; 18 male. m, masculine; f, feminine; control, control group; training, training group; ys, years; kg, kilogram; m, meters; h, hours*.

### Spontaneous sway

**Figure 4:**
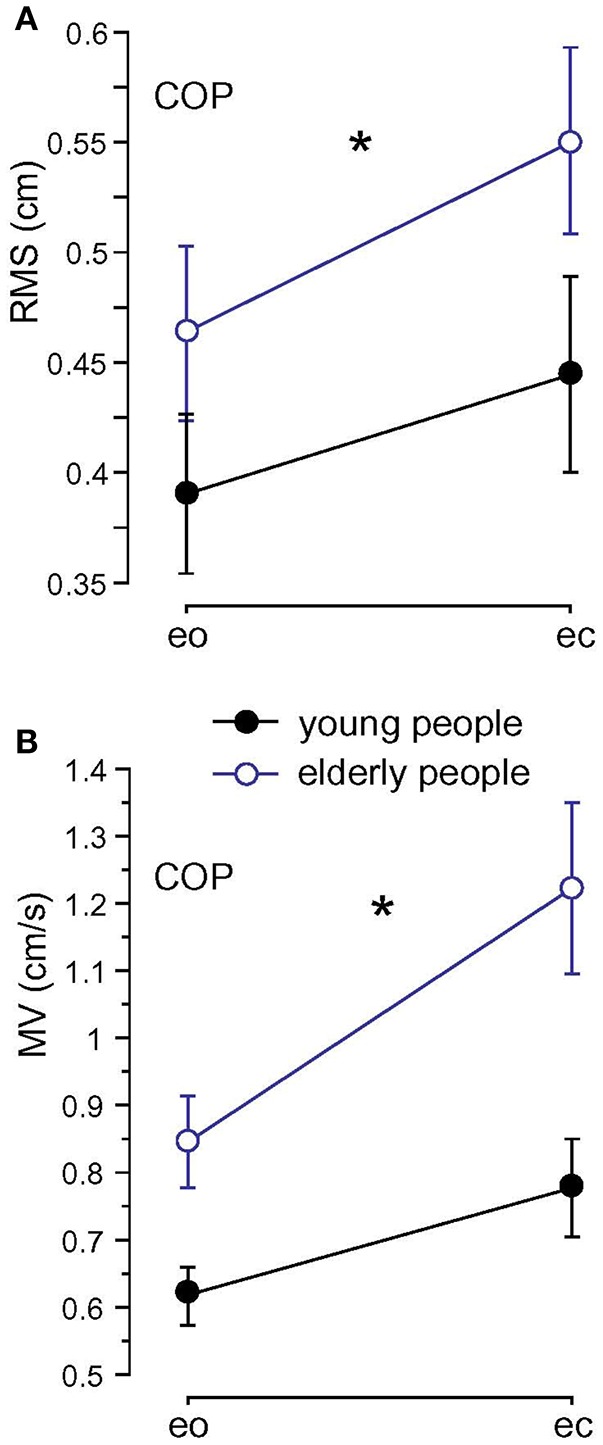
Spontaneous sway measures computed from center of pressure (COP) traces. **(A)** Root Mean Square (RMS). **(B)** Mean Velocity (MV) of the two age groups; eo, eyes open; ec, eyes closed. ^*^ Statistically significant difference (*p* < 0.05).

Root Mean Square (0.51 vs. 0.42 cm; *F* = 23.4, *p* < 0.001), MV (1.03 vs. 0.70 cm/s; *F* = 60.9, *p* < 0.001), and MF (0.49 vs. 0.41 Hz; *F* = 20.5, *p* < 0.001) were significantly larger in elderly before training (A1) compared to younger subjects (see Figure 4). We found no significant interactions between age and visual condition (*F* = 0.8, *p* = 0.39), sway direction (mediolateral, anteroposterior; *F* = 1.8, *p* = 0.18), and body segments (*F* = 1.9, *p* = 0.16). None of the measures significantly interacted with balance training for the elderly (RMS: *F* = 2.6, *p* = 0.11, MV: *F* = 1.7, *p* = 0.20, MF: *F* = 0.05, *p* = 0.83).

### Externally perturbed stance

#### GAIN

**Figure 5:**
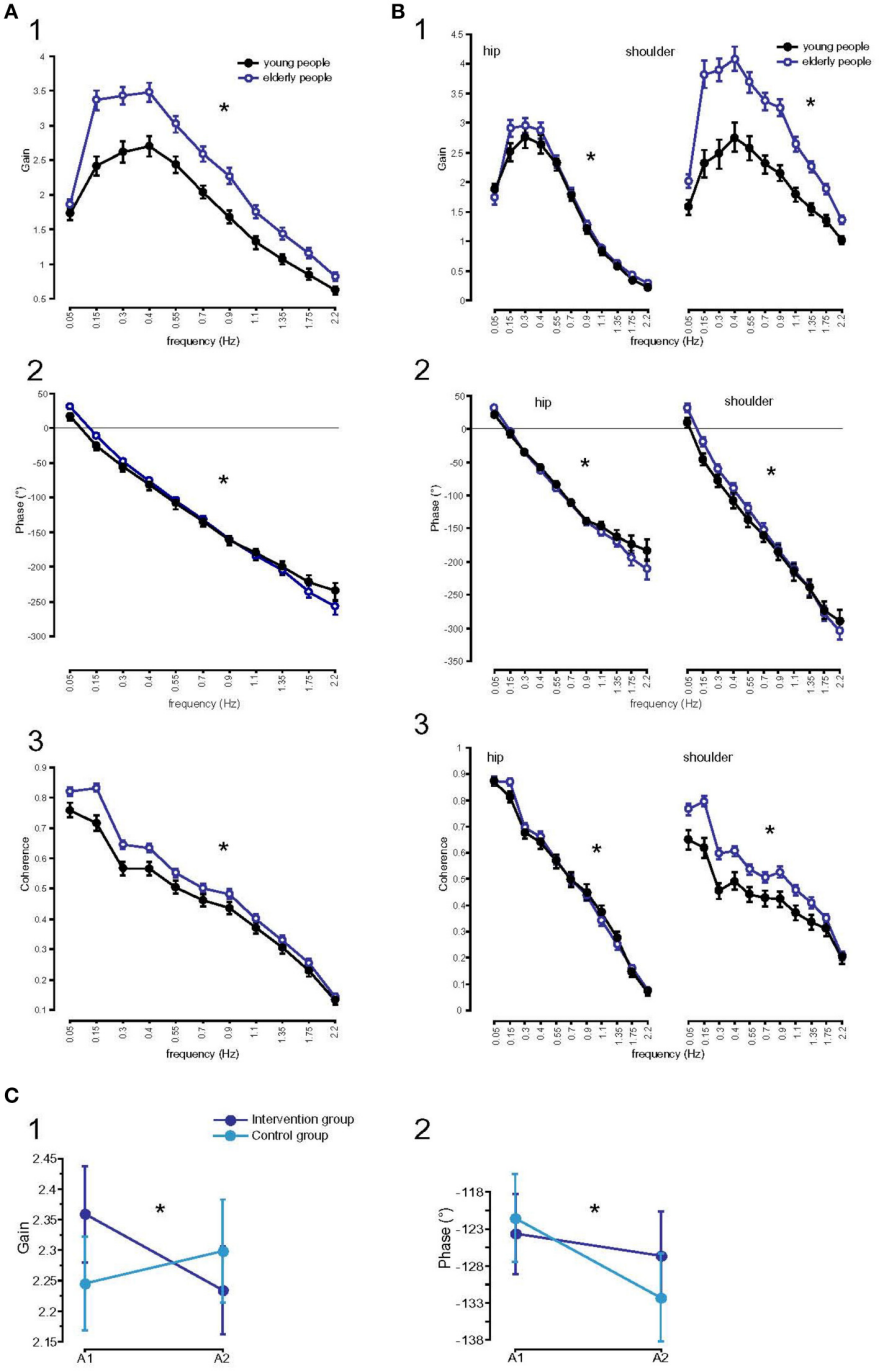
Parameters of perturbed stance analyzed at 11 frequencies. **(A)** GAIN (1), PHASE (2), and Coherence (3) of the two age groups across all stimulus amplitudes, body segments, and visual conditions. ^*^ Statistically significant difference (*p* < 0.05). °Degree; Hz, Hertz. **(B)** GAIN, PHASE, and Coherence, interactions between age and body segments. GAIN (1), PHASE (2), and Coherence (3) of the two age groups across all stimulus amplitudes and visual conditions separated by body segments. ^*^ Statistically significant difference (*p* < 0.05). °Degree; Hz, Hertz. **(C)** Influence of balance training on parameters of perturbed stance. GAIN (1) and PHASE (2) curves of the training and control group before (A1) and after (A2) training. ^*^Statistically significant difference (*p* < 0.05). °Degree.

In elderly subjects before training, GAIN was significantly larger (2.31; *F* = 553.7, *p* < 0.001) than in young subjects (1.77). Across the age groups, GAIN was significantly larger with eyes closed than with eyes open (eyes closed, ec: 2.36, eyes open, eo: 1.72; *F* = 766.7, *p* < 0.001). Stimulus amplitudes (0.5°: 2.24, 1°: 1.84; *F* = 307.4, *p* < 0.001), stimulus frequencies (*F* = 4954.3, *p* < 0.001), and body segments (hip: 1.60, shoulder: 2.48, *F* = 1482.5, *p* < 0.001) significantly influenced GAIN. Age group significantly interacted with frequency (*F* = 12.7, *p* < 0.001), with the most prominent GAIN difference between age groups in the lower frequency range (see GAIN plots in Figure 5A1). Moreover, we found a significant interaction between age group and body segments (*F* = 379.1, *p* < 0.001). This exemplifies that elderly subjects' shoulder GAIN (2.98) was almost twice as large as hip GAIN (1.64), whereas, young subjects' shoulder GAIN (1.99) was 20% larger than hip GAIN (1.55, Figure 5B1). Lastly, age group did not significantly interact with visual condition (*F* = 3.3, *p* = 0.07) or stimulus amplitude (*F* = 0.7, *p* = 0.41).

GAIN as a function of time (A1/A2) significantly interacted with balance training (*F* = 25.4, *p* < 0.001, Figure 5C1). While GAIN of the training group significantly decreased from 2.36 to 2.23 (*p* < 0.05), GAIN of the control group slightly increased (A1: 2.26, A2: 2.31, *p* > 0.05). Frequency did not interact significantly with GAIN as a function of time (*F* = 0.4, *p* = 0.95). However, GAIN as a function of time significantly interacted with body segments (*F* = 5.2, *p* = 0.02): Whereas GAIN of the shoulder decreased over time (2.97 to 2.90), GAIN of the hip hardly changed as a function of time (1.64 to 1.65). The decrease in shoulder GAIN is an effect of balance training. Shoulder GAIN of the training group decreased from 3.0 to 2.8, whereas shoulder GAIN of the control group slightly increased from 2.9 to 3.0 (*F* = 17.9, *p* < 0.001). In both groups, GAIN of the hip was nearly equal as a function of time (training group A1: 1.70, A2: 1.71; control group A1: 1.57, A2: 1.59). There were no significant interactions between time and visual condition (*F* = 0.4, *p* = 0.54), and between time and stimulus amplitude (*F* = 0.08, *p* = 0.78).

#### Phase

PHASE, indicating the temporal relationship between response and stimulus, differed significantly between the age groups (young subjects: −127.27°, elderly subjects: −122.34°; *F* = 8.9, *p* = 0.003). Across the age groups, PHASE was mainly determined by frequency, showing a PHASE lead in the low frequency range (*F* = 1035.9, *p* < 0.0001). In general, the significant interaction between age and frequency (*F* = 4.1, *p* < 0.001) showed the effect of age on PHASE as a function of frequency. The young group showed a moderate slope of PHASE as a function of stimulus frequencies, whereas the elderly group displayed a steeper relationship between PHASE and frequencies (see Figure 5A2). PHASE lag was found to be significantly smaller with eyes closed (−120.63°) than with eyes open (−128.99°, *F* = 25.7, *p* < 0.001), significantly smaller at the hip (−101.07°) than at the shoulder level (−148.54°, *F* = 828.6, *p* < 0.001) across all age groups. It did not significantly vary with different stimulus amplitudes (*F* = 0.009, *p* = 0.9). We found a significant interaction between age group and body segment (*F* = 45.2, *p* < 0.001) representing the fact that PHASE difference between shoulder and hip decreases with age (Figure 5B2). Age group did not significantly interact with visual condition (*F* = 1.6, *p* = 0.2) or stimulus amplitude (*F* = 0.6, *p* = 0.5).

PHASE lag as a function of time significantly interacted with balance training (*F* = 5.3, *p* = 0.02, Figure 5C2). Both, PHASE lag of the training group (−123.67° to −126.69°, *p* > 0.05) and PHASE lag of the control group (−120.94° to −131.70°, *p* < 0.05) increased as a function of time, with the increase being more pronounced in the control group. Time did not interact significantly with frequency (*F* = 0.1, *p* = 1.00). In addition, PHASE as a function of time significantly interacted with body segments (*F* = 22.1, *p* < 0.001). Whereas, PHASE lag of the hip was nearly stationary over time (−104.1 to −103.1), PHASE lag of the shoulder increased as a function of time (−140.5 to −155.3). However, we found no significant interaction between balance training and body segments as a function of time (*F* = 1.0, *p* = 0.3).

#### Coherence

In both, young and elderly subjects, coherence significantly depended on frequency (higher coherence with lower frequencies, *F* = 931.9, *p* < 0.001, Figure 5A3), stimulus amplitude (higher coherence with larger stimulus amplitude, *F* = 908.6, *p* < 0.001), on body segments (hip 0.49, shoulder 0.48; *F* = 7.5, *p* = 0.006), but not on visual condition (*F* = 0.6, *p* = 0.4). The coherence of the elderly group (0.52) was significantly higher than the coherence of the young group (0.46; *F* = 217.2, *p* < 0.001). There were significant interactions between age group and frequency (larger coherence differences between groups with lower frequencies, *F* = 5.8, *p* < 0.001), between age group and body segment (larger shoulder than hip coherence in elderly, smaller shoulder than hip coherence in young subjects, *F* = 142.9, *p* < 0.001, Figure 5B3). Age group did not significantly interact with stimulus amplitude (*F* = 0.4, *p* = 0.5) and coherence did not significantly interact with balance training (*F* = 0.1, *p* = 0.77).

### Model parameters (see Figures 6A–C)

**Figure 6:**
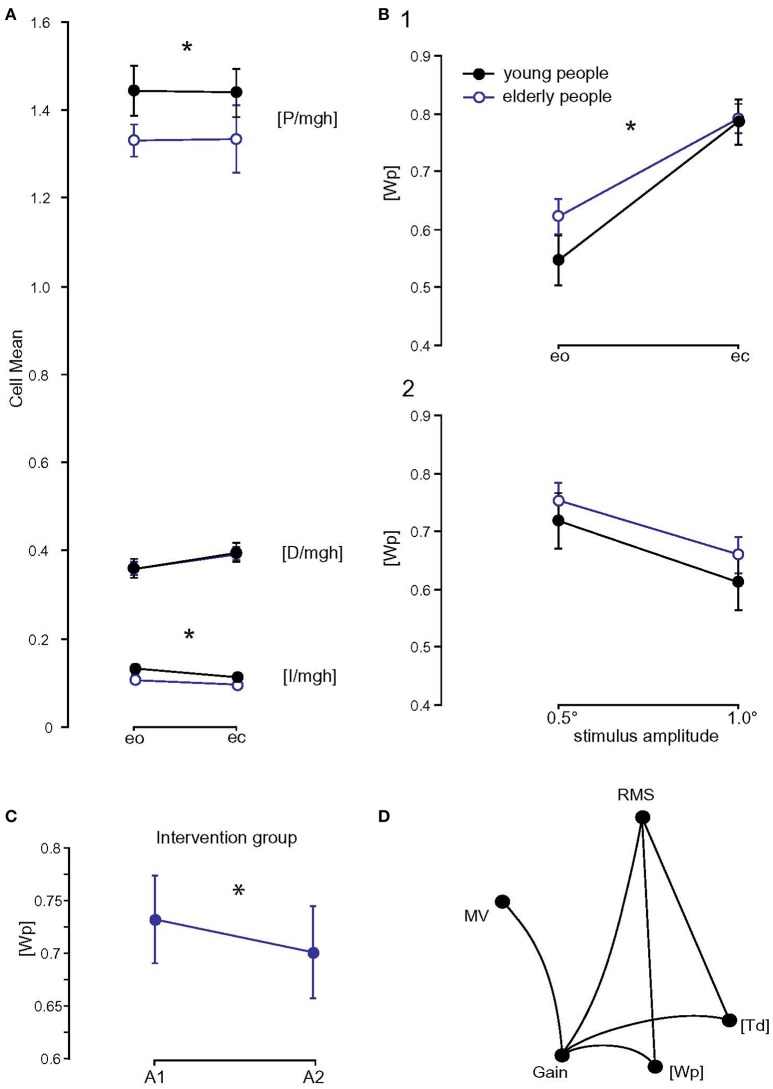
Model parameters and correlation matrix. **(A)** Model parameters of the two age groups ([P/mgh] in rad^−1^, [D/mgh] in s·rad^−1^, and [I/mgh] in s^−1^·rad^−1^); eo, eyes open; ec, eyes closed. ^*^ Statistically significant difference (*p* < 0.05). **(B)** [Wp] (proprioceptive sensory weight) of the two age groups with respect to visual condition (1) and stimulus amplitude (2); eo, eyes open; ec, eyes closed. ^*^ Statistically significant difference (*p* < 0.05). **(C)** Influence of balance training on [Wp]. [Wp] of the training group before (A1) and after (A2) balance training. **(D)** Correlation matrix of measures (spontaneous sway and perturbed stance measures) and parameters that differed significantly between young and elderly subjects. Only significant correlations are shown. RMS, Root Mean Square; MV, Mean Velocity; MF, Mean Frequency; [Wp], proprioceptive sensory weight; [Td], time delay.

The integral gain, [I/mgh], was significantly higher in the young group (0.12 s^−1^·rad^−1^) than in the elderly group (0.10 s^−1^·rad^−1^; *F* = 35.0, *p* < 0.001). It was significantly higher with eyes open (0.12 s^−1^·rad^−1^) than with eyes closed (0.10 s^−1^·rad^−1^; *F* = 20.5, *p* < 0.001). In addition, [Td] was significantly larger in elderly (0.17 s) compared to young subjects (0.16 s; *F* = 19.4, *p* < 0.001). Moreover, [Wp] was significantly larger in elderly compared to young subjects (0.71 vs. 0.67; *F* = 5.8, *p* = 0.016, Figure 6B). It was significantly larger with eyes closed than with eyes open (0.79 vs. 0.58; *F* = 143.3, *p* < 0.001) and it was larger at a stimulus amplitude of 0.5° (0.74) than at 1° (0.64; *F* = 34.5, *p* < 0.001). Age group significantly interacted with visual condition (*F* = 4.3, *p* = 0.04). The difference of [Wp] between the eyes-open and eyes-closed condition was greater in young (0.24) than in elderly subjects (0.17). We found no significant interaction between age group and stimulus amplitude. The derivative gain, [D/mgh], was not significantly different between the age groups (elderly subjects: 0.376 s·rad^−1^, young subjects: 0.378 s·rad^−1^; *F* = 0.06, *p* = 0.8). The proportional gain, [P/mgh], was significantly lower in elderly subjects (1.33 vs. 1.44 rad^−1^ in young subjects; *F* = 10.7, *p* = 0.001) and significantly lower at a stimulus amplitude of 0.5° (1.35 vs. 1.43 rad^−1^ at 1°; *F* = 6.3, *p* = 0.013). Passive stiffness, [Ppas], and passive damping, [Dpas], were significantly larger in young subjects compared to elderly subjects ([Ppas], young: 89.4, elderly: 84.4; *F* = 15.1, *p* = 0.001; [Dpas], young: 60.3, elderly: 57.4; *F* = 8.5, *p* = 0 .004). In general, [Ppas] und [Dpas] were larger with eyes open ([Ppas], eo: 92.9, ec: 80.9; *F* = 78.1, *p* < 0.001; [Dpas], eo: 61.6, ec 56.1; *F* = 32.5, *p* < 0.001). Visual condition and age group significantly interacted (*F* = 4.1, *p* = 0.042). The difference in [Ppas] between eyes-open and eyes-closed was greater in young (14.5) than in elderly subjects (10.7). Stimulus amplitude did not have a significant effect on [Ppas] (*F* = 2.6, *p* = 0.1).

Balance training did not have a significant effect on most model parameters ([I/mgh]: *F* = 0.004, *p* = 0.5, [P/mgh]: *F* = 0.8, *p* = 0.4, [D/mgh]: *F* = 1.3, *p* = 0.3, [Ppas]: *F* = 1.3, *p* = 0.3, [Dpas]: *F* = 0.3, *p* = 0.6, [Td]: *F* = 0.0, *p* = 1.0). However, the proprioceptive sensory weight, [Wp], changed significantly as a function of time (*F* = 4.0, *p* = 0.048, Figure 6C). [Wp] of the training group decreased from 0.73 to 0.70 (*p* < 0.05), [Wp] of the control group increased from 0.69 at the first assessment to 0.70 at the second assessment (*p* > 0.05).

### Clinical tests

The average reach distance of the training group increased significantly from 28.24 cm before to 32.08 cm after training (*F* = 7.4, *p* = 0.01). The reach distance of the control group decreased from 31.40 cm (A1) to 29.92 cm (A2) without being significant (*F* = 0.9, *p* = 0.4). Data of the TUG of the training group decreased during training but was not significant (A1: 8.48 s, A2: 8.34 s; *F* = 0.2, *p* = 0.7). Similar to the training group, there was no significant change in the TUG of the control group as a function of time (A1: 8.27 s, A2: 8.02 s; *F* = 0.2, *p* = 0.6).

### Correlations

A correlation matrix was computed between measures (spontaneous sway and perturbed stance measures) and parameters that differed significantly between young and elderly subjects. Spontaneous sway measures RMS (*r* = 0.56, *p* = 0.0005) and MV (*r* = 0.42, *p* = 0.013), significantly correlated with GAIN. RMS also correlated with [Wp] (*r* = 0.36, *p* = 0.03). GAIN correlated with [Wp] (*r* = 0.37, *p* = 0.03) and [Td] (*r* = 0.45, *p* = 0.008, see Figure 6D).

## Discussion

Here, the effect of balance training on postural control in elderly people was analyzed using a disturbance-related reactive motor approach. Postural control was assessed by spontaneous sway measures and measures of externally perturbed stance. Stimulus-response data were interpreted using a systems analysis approach (Engelhart et al., 2014; Pasma et al., 2014; Wiesmeier et al., 2015). We hypothesized that elderly subjects' postural control differed from that of young subjects, and that it was modified by balance training toward young subjects' postural control. In fact, elderly subjects displayed larger spontaneous sway amplitudes, velocities, and larger postural reactions than young subjects. Balance training reduced postural reaction sizes, which approached the range of values of young subjects. Using parameter identification techniques based on brain network model simulations, we found that balance training reduced overactive proprioceptive feedback and restored vestibular orientation in elderly. In the next paragraphs, we discuss the main findings sorted by the parameters analyzed, starting with age effects and followed by training effects, respectively.

Spontaneous sway was assessed using amplitude-related (RMS), velocity-related (MV), and frequency-related (MF) measures. All these measures have been reported to be higher in elderly than in young people (e.g., Prieto et al., 1996; Maurer and Peterka, 2005). In the present study, these differences were reproduced consistently.

While some authors reported effects of elderly's balance training on spontaneous sway (Judge, 2003; Hue et al., 2004; Nagy et al., 2007), we did not find significant effects. Some researchers interpreted smaller postural sway as improved balance (Judge, 2003; Hue et al., 2004). Others interpreted increased sway after balance training as an improved balance due to increased confidence (Nagy et al., 2007). As discussed in recent papers, different postural control deficits might lead to similar abnormalities in spontaneous sway measures reducing its usability for specific assessments of balance (Ghulyan et al., 2005; Wiesmeier et al., 2015).

Subjects' postural reactions as a function of external perturbations, i.e., anterior-posterior platform tilts, were characterized using GAIN and PHASE curves. Similar to reports in earlier papers, elderly subjects' postural reactions, i.e., GAIN values, were larger than in young subjects. This effect was more pronounced at the shoulder than at the hip level (Ghulyan et al., 2005; Wiesmeier et al., 2015). In other words, elderly subjects were dragged with the platform, while young subjects were more stable in space.

Balance training significantly reduced GAIN values toward the values of young subjects. The benefit of training amounted to about 30% of the GAIN difference between elderly and young subjects. If we assume a linear relationship between deterioration of postural control and age, based on former studies (Era et al., 2006; Wiesmeier et al., 2015), the training effect could be extrapolated as a juvenescence of about 10 years given the average age difference of 36 years between the two age groups.

In general, larger postural reactions in elderly could be due to an intensified use of ankle proprioception and a reduced use of vestibular information. Vestibular information would be used to stabilize the body in space. Other reasons for large postural reactions might include general muscle weakness, or an increased time delay between stimulus and response. In order to separate the different possible subsystems responsible for increased postural reactions, we applied a systems analysis approach based on a well-known postural control model (Engelhart et al., 2014; Pasma et al., 2014).

Using model simulations, we were in fact able to identify larger contributions of proprioception to sensory feedback in elderly as compared to young subjects. The higher the proprioceptive feedback, the lower the contributions of space cues, i.e., the vestibular information, when eyes were closed, and vestibular and visual information with eyes open. With a decreased vestibular feedback, elderly people are closer to vestibular loss patients (see in Maurer et al., 2006) than to patients suffering from polyneuropathy (unpublished data). As we showed before (Maurer et al., 2006), vestibular loss patients, who are forced to rely 100% on proprioception, tend to fall on tilting platforms, signifying the problems with a pure proprioceptive strategy.

After training, the proprioceptive feedback was significantly reduced with both eyes open and eyes closed. The decrease of proprioceptive feedback in the eyes-closed condition can only be explained by an increase of the vestibular feedback. This indicates that elderly subjects learned to weigh vestibular information higher. Because the increase of the weight for space cues is similar in the eyes-open condition, we assume that this, again, is caused by an increase of the vestibular feedback. Interestingly, proprioceptive feedback was not only the most prominent difference between elderly and young subjects, but also the only parameter that was affected by training. The other parameters, that differed between young and elderly subjects (larger time delay [Td], smaller integral gain [I/mgh], smaller proportional gain [P/mgh], smaller passive stiffness factor [Ppas], and smaller passive damping factor [Dpas] in elderly), were not significantly affected by balance training. This could be due to the fact that the parameters not affected by training represent those physiological constituents of postural control that may be closely related to anatomical features of the subject (Peterka, 2002), such as, height of the COM, mass distribution, patterns of muscle recruitment, or nerve conduction time. While balance training could principally affect plastic central weighting processes, it is less likely that they directly influence anatomical constraints of the body.

Additional differences between young and elderly subjects' postural reactions were related to a more pronounced PHASE slope and a smaller PHASE lag (Ghulyan et al., 2005; Wiesmeier et al., 2015). More specifically, PHASE difference between shoulder and hip decreased with age, pointing to a different coordination of body segments. We interpreted this as a change in reactive balance strategy using more hip flexion and extension. This effect might be in accordance to a hypothesis presented by Kuo et al. (1998) that the use of hip flexion/extension may be enhanced in conditions where the support surface is not reliable, i.e., in unstable platform conditions. All these additional differences were not significantly affected by balance training.

Experimental results were compared with known clinical tests for the assessment of postural deficits, namely the FRT and the TUG TEST (Enkelaar et al., 2013). The FRT was significantly ameliorated by balance training. This is in accordance with the expected effects of balance training, as FRT clinically stands for the ability to balance. FRT values of our elderly group were similar to the ones reported by Duncan et al. (1990) who assessed 128 volunteers between 21 and 87 years. The TUG was improved after balance training (not significantly). The TUG scores of the training and control groups corresponded to scores reported by Nagy et al. (2007; 8.9–10.3 s) and Enkelaar et al. (2013; 9.3 s).

The correlation analysis of elderly's data before and after training revealed that the significant effect of balance training, represented by the larger postural reactions (GAIN) significantly correlated with the strength of proprioceptive feedback [Wp], with RMS, with MV, and with time delay [Td]. This correlation pattern indicates that there is a certain tendency that the elderly ameliorated spontaneous sway measures and time delay with training, which is correlated with the main training effect of the recovery of vestibular function.

## Conclusion

Balance training reduced elderly subjects' overactive proprioceptive feedback and enhanced vestibular orientation. The modified use of sensory information can be interpreted as a change in postural control strategies representing a higher level adaptive mechanism. Based on the assumption of a linear deterioration of postural control across the life span, the training effect can be extrapolated as a juvenescence of about 10 years. This is even more surprising, given the fact that the elderly subjects evaluated here were in a healthy and active state prior to the study. We hold that this study points to a considerable benefit of a continuous balance training in elderly, even without any sensorimotor deficits.

## Ethics statement

This study was carried out in accordance with the recommendations of Ethics Committee of Freiburg University with written informed consent from all subjects. All subjects gave written informed consent in accordance with the Declaration of Helsinki. The protocol was approved by the Ethics Committee of Freiburg University.

## Author contributions

IW and CM: substantial contributions to the conception and design of the study, data acquisition, analysis, and interpretation, drafted and revised manuscript, DD: substantial contributions to the conception and design of the study, acquisition, analysis, and interpretation of data, AW and JD: conception and design of the study, data acquisition, revised manuscript, UG, TM, CW, and AG: substantial contributions to the conception and design of the study, data acquisition and interpretation, revised manuscript.

### Conflict of interest statement

The authors declare that the research was conducted in the absence of any commercial or financial relationships that could be construed as a potential conflict of interest.
