# Supplementary material for: Balance Training Enhances Vestibular Function and Reduces Overactive Proprioceptive Feedback in Elderly
Source: Front Aging Neurosci. 2017 Aug 11;9:273. doi: 10.3389/fnagi.2017.00273 (PMC5554492; doi:10.3389/fnagi.2017.00273)

Supplementary Material

Balance training enhances vestibular function and reduces overactive proprioceptive feedback in elderly

Isabella Katharina Wiesmeier, Daniela Dalin, Anja Wehrle, Urs Granacher, Thomas Muehlbauer, Joerg Dietterle, Cornelius Weiller, Albert Gollhofer, Christoph Maurer *

*** Correspondence:** Corresponding Author: christoph.maurer@uniklinik-freiburg.de

# Goodness of Fit

To investigate the goodness of the model fits, the goodness of fit (GOF) in the frequency domain was calculated as follows:

| $GOF=\left[ 1-\frac{\sum_{k=1}^{N} \left\vert S_{fit}\left( f_{k} \right)-S_{exp}\left( f_{k} \right) \right\vert^{2}}{\sum_{k=1}^{N} \left\vert S_{fit}(f_{k}) \right\vert^{2}} \right]*100\%$ |  |
| --- | --- |

S_exp_ and S_fit_ are the experimental and fitted sensitivity functions, respectively. N is the number of excited frequencies, (f) the excited frequencies. The goodness of the balance control model fits was assessed by the GOF. The mean (± SD) GOF for the elderly subjects in the 0.5 and 1.0 degree peak-to-peak condition was 82 (± 11) % and 85 (± 6) %, respectively. Likewise, the GOF values for the young subjects in the 0.5 and 1.0 degree peak-to-peak condition were 78(± 15) % and 88 (± 7) %.

# Comparison to simulations of datasets from other studies

The core mechanisms of the model used here are also part of other studies, all mentioned in the main manuscript, see also list below.

- Cenciarini M, Loughlin PJ, Sparto PJ, Redfern MS. Stiffness and damping in postural control increase with age. IEEE Transactions on Bio-Medical Engineering 2010;57(2):267–275. doi:10.1109/TBME.2009.2031874
- Engelhart D, Pasma JH, Schouten AC, et al. Impaired standing balance in elderly: a new engineering method helps to unravel causes and effects. Journal of the American Medical Directors Association 2014;15(3):227.e1–6. doi:10.1016/j.jamda.2013.09.009
- Maurer C, Mergner T, Peterka RJ. Multisensory control of human upright stance. Experimental Brain Research 2006;171(2):231–250. doi:10.1007/s00221-005-0256-y
- Maurer C, Peterka RJ. A new interpretation of spontaneous sway measures based on a simple model of human postural control. Journal of Neurophysiology 2005;93(1):189–200. doi:10.1152/jn.00221.2004
- Pasma JH, Engelhart D, Schouten AC, van der Kooij H, Maier AB, Meskers CG. Impaired standing balance: the clinical need for closing the loop. Neuroscience 2014; 267:157-165. doi: 10.1016/j.neuroscience.2014.02.030. Epub 2014 Mar 6.
- Peterka RJ. Sensorimotor integration in human postural control. Journal of Neurophysiology 2002;88(3):1097–1118. doi:10.1152/jn.00605.2001
- Van der Kooij H, Peterka RJ. Non-linear stimulus-response behavior of the human stance control system is predicted by optimization of a system with sensory and motor noise. Journal of Computational Neuroscience 2011; 30(3):759–778. doi:10.1007/s10827-010-0291-y
- Wiesmeier IK, Dalin D, Maurer C. Elderly use proprioception rather than visual and vestibular cues for postural motor control. Front. Aging Neurosci. 2015;7:97. doi: 10.3389/fnagi.2015.00097

Slight differences mainly relate to additional filters for motor output and force feedback, which were not used in this study.

# Limitations of the model

In addition to the assumptions explained in the methods section (sensory feedback signals add up to a gain of unit, all gains are constrained to positive values), the models mentioned above rely on the assumption that the sensory input is continuously fed back to generate motor output. There are other modeling approaches which assume a more pulsatile motor correction. Moreover, the authors listed above use linearized postural control models. Others use non-linear approaches based on perception thresholds, or prediction. The reason for using this type of model is due to the fact that one cannot identify more than 5-6 different model parameters without performing more extended experiments like e.g. testing many more stimulus amplitudes, or measuring individual perception thresholds etc.

# Questionnaire

Here we provide the questionnaire for the elderly subjects (in German).


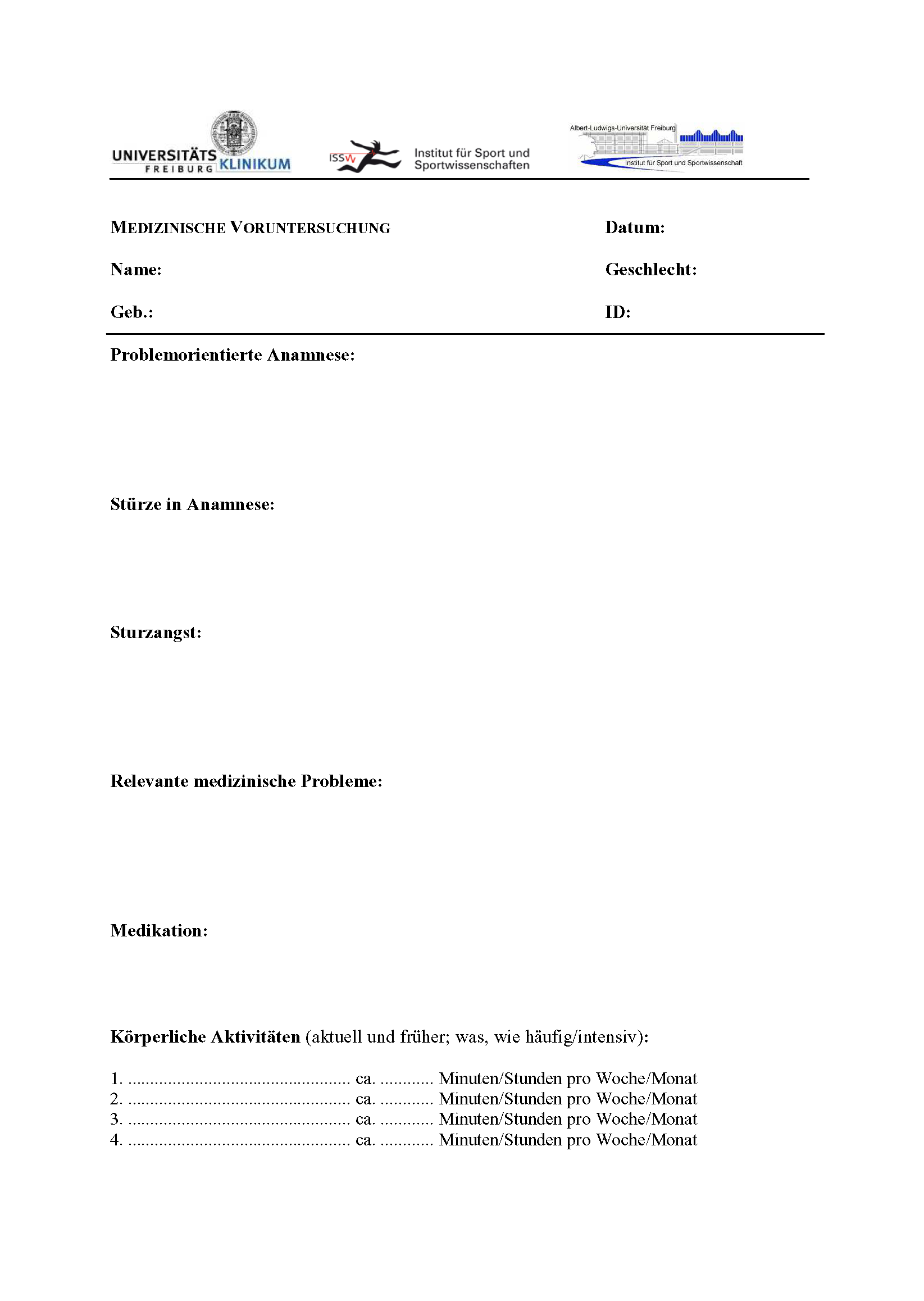

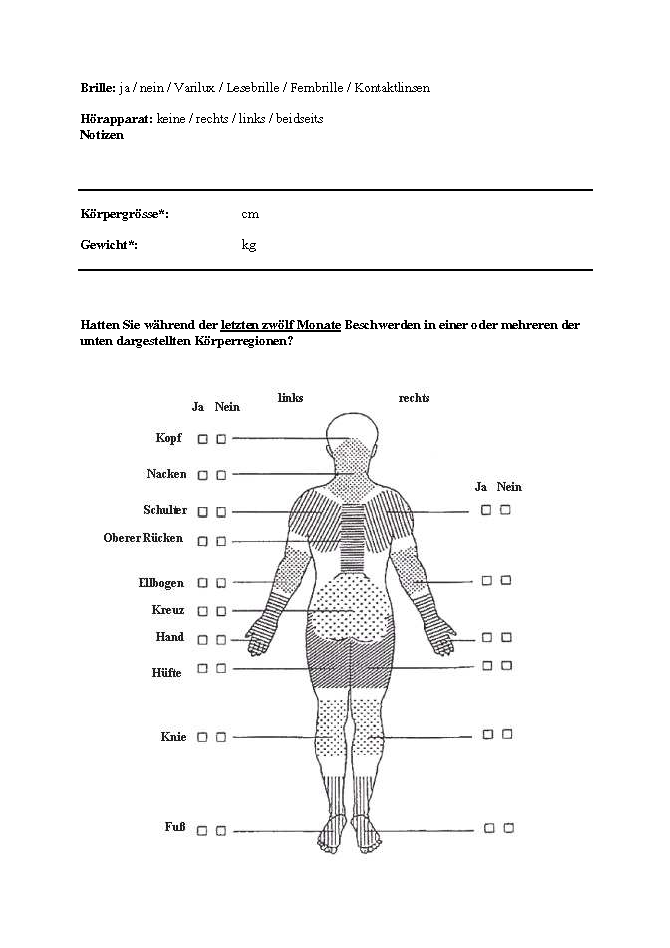

Supplement: Supplementary file 1 [file DataSheet1.DOCX]
